# Prognostic Value of Atherosclerotic Extent in Diabetic Patients with Nonobstructive Coronary Artery Disease

**DOI:** 10.1155/2021/5597467

**Published:** 2021-06-09

**Authors:** Yipu Ding, Zinuan Liu, Guanhua Dou, Xia Yang, Xi Wang, Dongkai Shan, Bai He, Jing Jing, Yundai Chen, Junjie Yang

**Affiliations:** ^1^Department of Cardiology, Chinese PLA General Hospital, Beijing 100853, China; ^2^School of Medicine, Nankai University, Tianjin 300071, China

## Abstract

**Methods and Results:**

813 DM patients (mean age 58.9 ± 9.9 years, 48.1% male) referred for CCTA due to suspected CAD in 2015-2017 were consecutively included. During a median follow-up of 31.77 months, 50 major adverse cardiovascular events (MACEs) (6.15%) were experienced, including 2 cardiovascular deaths, 14 nonfatal myocardial infarctions, 27 unstable anginas requiring hospitalization, and 7 strokes. Three groups were defined based on coronary stenosis combined with Leiden score as normal, nonobstructive Leiden < 5, and nonobstructive Leiden ≥ 5. Cox models were used to assess the prognosis of plaque burden within these groups. An incremental incidence of MACE rates was observed. After adjustment for age, gender, and presence of high-risk plaque, the group of Leiden ≥ 5 showed a higher risk than Leiden < 5 (HR: 1.88, 95% CI: 1.03-3.42, *p* = 0.039). Similar results were observed when segment involvement score (SIS) was used for sensitivity analysis.

**Conclusion:**

Atherosclerotic extent was associated with the prognosis of DM patients with nonobstructive coronary artery disease, highlighting the importance of better risk stratification and management.

## 1. Introduction

It is well established that diabetes mellitus (DM) is associated with coronary artery disease (CAD) and a higher rate of mortality [1]. In turn, the rising prevalence of coronary artery disease, along with increased ischemic events, represents an important cardiac threat to DM patients. Early detection of CAD in this population has been an urgent requirement for the primary and secondary prevention of both fatal and nonfatal cardiac events [2–4].

Although there is no clear evidence suggesting the imaging evaluation of CAD in DM patients [5], the current practice guideline stands that coronary computed tomography angiography should be an access to cardiac risk assessment in the presence of DM with its high accuracy and acceptance [6]. Previous studies have shown that atherosclerotic extent derived by coronary computed tomography angiography (CCTA) has an extraordinary ability in risk stratification among nonobstructive CAD patients, to which little attention was paid due to the moderate stenosis [7]. However, few researches have been conducted on DM patients, despite the higher risk of major adverse cardiovascular events among them. Using comprehensive risk scores as a quantitative index, we aimed to investigate the stratification capability of atherosclerotic extent in DM patients with nonobstructive CAD.

## 2. Materials and Methods

### 2.1. Patients

This study was approved by the local ethics committee, and informed consent was obtained from all participants. Between Jan 1, 2015, and Dec 31, 2017, 2135 DM patients who had undergone CCTA for suspected CAD in our institution were prospectively enrolled. Patients with known CAD, a history of percutaneous coronary intervention or coronary bypass surgery, a history of myocardial infarction or myocarditis, or revascularization driven by CCTA results within 3 months were excluded. Those with incomplete baseline data or uninterpretable CCTA results were ruled out of further analysis. In addition, only mild lesion was our concern, so the obstructive CAD was excluded according to CCTA definition mentioned below.

Basic demographic data were obtained by a review of medical records or patient interviews. DM was defined as fasting blood glucose ≥ 7.0 mmol/L or 2 h plasma glucose ≥ 11.1 mmol/L during oral glucose tolerance test or A1C ≥ 6.5% (48 mmol/mol) or the use of oral hypoglycemic agents/insulin. The following cardiac risk factors were recorded: (1) hypertension (a systolic blood pressure ≥ 140 mmHg or a diastolic blood pressure ≥ 90 mmHg or administration of antihypertensive therapy), (2) hypercholesterolemia (known but untreated dyslipidaemia or current treatment with lipid-lowering medications), (3) positive family history of CAD (presence of CAD in first-degree relatives at <55 years in men and <65 years in women), and (4) smoking (current smoking or cessation of smoking within 3 months of CCTA).

### 2.2. Image Acquisition and Analysis

Multidetector CCTA scans were performed on a dual-source CT scanner (Somatom Definition Flash CT, Siemens Medical Solutions, Forchheim, Germany). All scans were analysed using a dedicated workstation (Syngo.via, Siemens) by two experienced cardiologists. When disagreements existed on diagnosis, the final decision would be made through consultation or the intervention of a third experienced researcher.

According to the modified American Heart Association classification, coronary lesions were assessed on the basis of the 17-segment model visually [8]. All segments were coded for the presence, composition, and severity of coronary plaque and were classified as normal, nonobstructive (1% to 49% luminal stenosis), or obstructive (>50% luminal stenosis). Calcified plaque was defined as having a density of >130 HU and further specified as “spotty” if its maximum diameter is <3 mm in any direction. Noncalcified plaque was defined as having an attenuation value lower than that of the contrasted vessel lumen. When both types existed, mixed plaque was defined. “Low CT attenuation plaques” were the presence of a central focal area within the plaque which has a low CT attenuation which is usually defined as at least 1 voxel with <30 HU. If the outer vessel diameter is >10% greater than the mean of the diameter of the normal adjoining segments, “positive remodelling” was recognized. “Napkin ring sign” was the presence of circumferential necrotic core. With at least two characteristics of “spotty calcification,” “low CT attenuation plaques,” “positive remodelling,” and “napkin ring sign”, high-risk plaque (HRP) was recorded [9, 10].

### 2.3. Comprehensive Risk Scores

**Figure 1:**
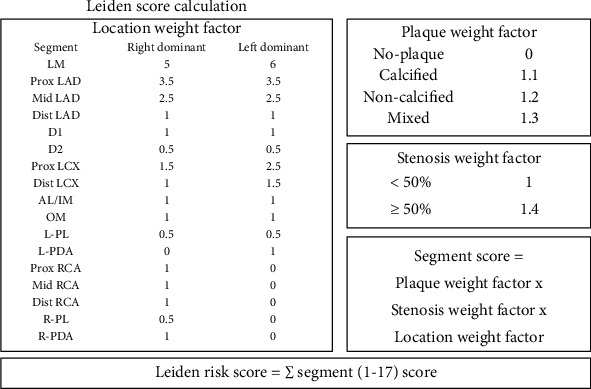
Schematic overview of the computed tomography angiography-derived risk score. Leiden score is calculated by the summation of segment score quantified as plaque weight factor × stenosis weight factor × location weight factor, i.e., a right dominant system with a noncalcified plaque with >50% stenosis in the left main segment (5 × 1.2 × 1.4) + a noncalcified plaque with <50% stenosis in the proximal left circumflex artery (1.5 × 1.2 × 1) + a calcified plaque with >50% stenosis in the right posterior descending artery (1 × 1.1 × 1.4), so the Leiden score is 11.74. Segment involvement score (SIS) was calculated by the summation of the segments exhibiting plaque; in the case above, SIS is 3. CTA = computed tomography angiography; AL = anterolateral segment; D1 = diagonal 1; D2 = diagonal 2; IM = intermediate segment; LAD = left anterior descending coronary artery; LCA = left coronary artery; LCX = left circumflex coronary artery; LM = left main segment; L-PDA = left posterior descending artery; L-PL = left posterolateral segment; OM = obtuse marginal segment; RCA = right coronary artery; R-PDA = right posterior descending artery; R-PL = right posterolateral segment.

Leiden score, a comprehensive risk score, was introduced as a quantitative index of atherosclerotic burden, containing information of plaque quantity, location, stenosis, and composition as shown in Figure 1. The segment involvement score (SIS) was obtained to quantify the atherosclerotic extent for sensitivity analysis, calculated as the total number of coronary artery segments that exhibits plaque without consideration of stenosis (ranging from 0 to 16).

### 2.4. Follow-Up and Study Endpoint

Follow-up information was obtained by phone contact or the electronic medical record system. The primary endpoint was cardiovascular death, nonfatal myocardial infarction, stroke, or unstable angina requiring hospitalization that occurred >90 days after the CCTA examination from Jan 1, 2015, to Aug 31, 2020. Each event was identified by two physicians independently. In the case of divergence, consultation would be brought in.

### 2.5. Statistical Analysis

Analyses were performed with SPSS version 26.0 (SPSS, IL, USA) and R version 3.6.3. Baseline characteristics were presented as mean ± standard deviation or median (interquartile range (IQR)) for continuous variables and as proportions for categorical variables. Prevalence of no or nonobstructive CAD was calculated and stratified by the comprehensive risk score as normal group (no CAD), nonobstructive CAD with Leiden < 5, and nonobstructive CAD with Leiden ≥ 5. Sensitivity analysis was conducted with SIS, stratifying patients as the normal group (no CAD), nonobstructive CAD with SIS < 3, and nonobstructive CAD with SIS ≥ 3. Cumulative event rates were estimated using the Kaplan-Meier method and compared using the log-rank test. Cox proportional regression model was used to investigate multivariable-adjusted hazard ratios for increasing CAD severity mentioned above. A *p* value less than 0.05 was considered as statistically significant.

## 3. Results

### 3.1. Baseline Characteristics

A total of 2135 DM patients who underwent CCTA for suspected CAD were enrolled, among which 51 were lost during follow-up. 1271 patients were excluded because of known CAD, revascularization, incomplete data, or other criteria. A cohort of 813 diabetic patients (mean age 58.9 ± 9.9 years; 48.1% male; median follow-up 31.77 months) was included with full demographic characteristic and CCTA information. The prevalence of hypertension, hypercholesterolemia, current smoking, and a family history of CAD was 64.8%, 54.4%, 24.2%, and 23.6%, respectively (Table 1). For glucose control, 19.7% of the patients solely had a diet, 80.9% had oral hypoglycemic medication, and insulin was used in 14.3% of the patients. Overall, 190 (23.4%) of the 813 patients had no evidence of CAD on coronary CTA. In addition, high-risk plaques were found in 18 (2.2%) patients.

### 3.2. Cox Regression Analysis

In univariate analysis, age (HR: 1.04, 95% CI: 1.01-1.07) and the presence of HRP (HR: 11.66, 95% CI: 5.45-24.95) were associated with MACEs. Compared with the normal group, HR was 1.86 (95% CI: 0.70-5.00, *p* = 0.216) for the group of nonobstructive Leiden < 5 and 4.06 (95% CI: 1.56-10.56, *p* = 0.004) for nonobstructive Leiden ≥ 5, respectively.

In multivariate models, age (HR: 1.03, 95% CI: 1.00-1.07) and HRP (HR: 10.94, 95% CI: 5.00-23.92) remained significant in predicting outcome events (Table 2). Patients with nonobstructive Leiden ≥ 5 had an unadjusted hazard ratio of 4.06 (95% CI: 1.56 to 10.56, *p* = 0.004; log-rank test: *p* = 0.0015) (Table 2). After adjustment for age, gender, and presence of HRP, the hazard ratio remained significantly higher, which was 2.94 (95% CI: 1.11 to 7.79, *p* = 0.031) and 1.88 (95% CI: 1.03 to 3.42, *p* = 0.039), in comparison to the normal group and nonobstructive Leiden < 5, respectively.

### 3.3. Survival Analysis

**Figure 2:**
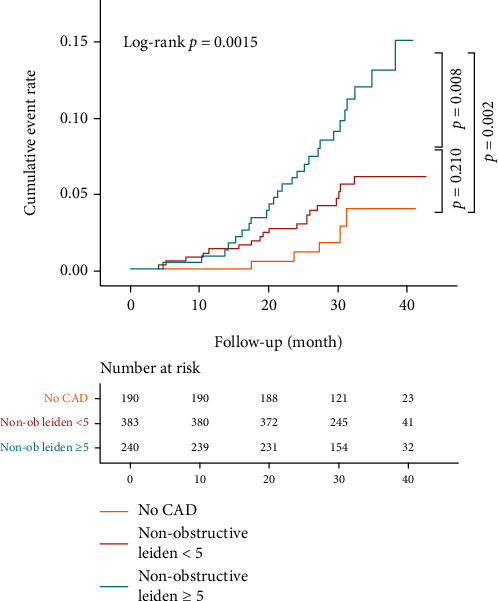
Cumulative risk of the composite endpoint on the basis of CAD severity with Leiden risk score (no CAD, nonobstructive CAD with Leiden < 5, and nonobstructive CAD with Leiden ≥ 5). CAD: coronary artery disease.

Of the included 813 patients, 50 MACEs (6.15%) were experienced, including 2 cardiovascular deaths, 14 nonfatal myocardial infarctions, 27 unstable anginas requiring hospitalization, and 7 strokes (Figure 2). The annual MACE rate among patients in the normal group was 0.98 events per100 person-years, and the annual MACE rate among nonobstructive Leiden < 5 was 1.86 per 100 person-years, while the rate for nonobstructive Leiden ≥ 5 was 4.06 per 100 person-years (*p* < 0.01).

### 3.4. Sensitivity Analysis

**Figure 3:**
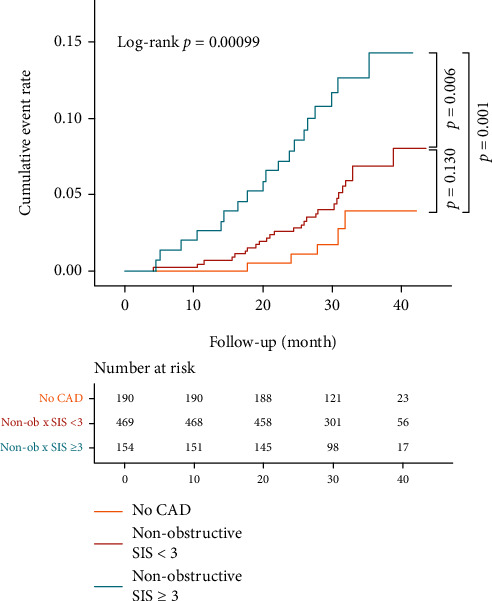
Cumulative risk of the composite endpoint on the basis of CAD severity with segment involvement score (no CAD, nonobstructive with SIS < 3, and nonobstructive with SIS ≥ 3). CAD: coronary artery disease; SIS: segment involvement score.

For further sensitivity analysis, segment involvement score (SIS) was used to quantify the atherosclerotic extent instead. A comparable distribution of event rate has been noticed (Figure 3), of which the normal group, the nonobstructive SIS < 3 group, and the nonobstructive SIS ≥ 3 group were 2.63%, 5.54%, and 12.34%, respectively. In the adjusted Cox model, patients with nonobstructive SIS ≥ 3 conferred a significantly higher risk than those in both the normal group (HR: 3.49, 95% CI: 1.28-9.52, *p* = 0.015) and the nonobstructive SIS < 3 group (HR: 2.14, 95% CI: 1.17-3.91, *p* = 0.013).

## 4. Discussion

The main finding of this study was that in DM patients with nonobstructive CAD, higher atherosclerotic extent on CCTA provided incremental prognostic information and was associated with long-term cardiovascular outcome, even after adjustment for traditional risk factors including age, gender, and high-risk plaque profiles. Our results reinforced the notion that greater efforts are needed to promote risk stratification with nonobstructive CAD, especially in the presence of DM. Leiden risk score represented an effective and reliable tool for quantifying atherosclerotic extent, which had a substantial impact on clinical outcome in diabetic patients. The robustness of the conclusion was further evaluated with the sensitivity analysis using SIS, with similar main results observed.

Our findings concur with a previous cohort study [11], which demonstrated that it is possible to identify high-risk diabetic patients based on assessment of CAD through CCTA. However, several disparities must be noted. A slightly higher MACE rate was presented, compared with an annual event rate ranging from 1.5% to 16.9% as a meta-analysis showed [1], in which diabetes examined by CCTA was investigated. One possibility is that we broadened enrollment to MACEs with strokes and an extended follow-up to a median of 31 months, which was a sufficient duration to capture more events. Moreover, up to 80% of the patients received hypoglycemic therapy in baseline, indicating a potentially long duration of diabetes and higher vascular risk. Another important observation from our study is that in risk-adjusted hazard analysis, the presence of HRP was found an independent predictor with a high HR of 3.15 (95% CI: 1.97-5.04). This corresponds with the result from the ICONIC study [12] that stressed the importance of HRP+ lesions in nonobstructive CAD, exhibiting a comparable risk of becoming a culprit lesion to obstructive HRP- lesions. In view of this, we bring it into analysis, which has rarely been studied before. However, after adjustment for HRP, extensive nonobstructive CAD was still found a significant indicator. This finding may inform future trials of the potential role of nonobstructive CAD in the setting of diabetes.

In the PROMISE (Prospective Multicenter Imaging Study for Evaluation of Chest Pain) trial, most cardiovascular deaths or myocardial infarctions (67%) occurred in patients with a normal stress test result at baseline, most of whom were found to have nonobstructive atherosclerotic disease by cardiac CT [13]. This suggests that we miss the opportunity to implement comprehensive preventive measures in most patients, especially in diabetic patients, by relying on stress test results. The SCOT-HEART (Scottish Computed Tomography of the Heart) trial revealed a reduction of 41% in the hazard of CAD-related death or nonfatal myocardial infarction for patients who were assigned to an anatomic versus functional strategy (2.4% vs. 3.9%) [14]. This was attributed to detection of nonobstructive coronary atherosclerosis and the initiation of directed preventive treatment. Our study was partly in line with the results above and further stressed the importance of medical management in diabetic patients with extensive nonobstructive coronary artery disease. The ability of noninvasively detecting nonobstructive atherosclerotic disease by CT, thus, should be rendered as a necessary opportunity to initiate earlier prevention or intensive treatment in the process of disease, a strategy proven effective in reducing MACEs [15].

Some previous studies have evaluated the extent and distribution of atherosclerosis with semiquantitative CCTA risk score in diabetes, mainly based on the SIS or the segment stenosis score (SSS) [16]. However, neither SIS nor SSS reflects the importance of relevant segment in coronary artery, because the proximal segment in the artery holds accountability for myocardial perfusion of larger territory. In this circumstance, the Leiden comprehensive risk score, being reported more strongly predictive than the SIS, integrates stenosis severity with the number and location of stenosis. A recent research from van den Hoogen et al. [17] evaluated the per-segment and per-patient weight scores to determine the contribution of the stenosis, composition, and location of CAD to the total score. As a result, all the per-patient weight scores were significantly higher in the setting of DM, while the per-segment location weight score was lower, which might be explained by the multisegment disease in DM patients. We also used SIS for sensitivity analysis to stratify the extent of atherosclerotic plaque, which demonstrated the similar result and further supported our hypothesis.

## 5. Study Limitation

First, as a retrospective single center study, referral decision for CCTA was made by physicians independently and certain patients were excluded finally due to various reasons, which may introduce selection bias. Second, diabetes is a dynamic risk factor; lack of the diabetes duration and treatment information on baseline may cause the misinterpretation of the subsequent data analysis. Third, although downstream treatment and management were recorded, relative treatments were not included in the final multivariate analysis, which may lead to potential confounders and over- or underestimation of the effect size of target variables.

## 6. Conclusion

In diabetic patients with nonobstructive CAD, atherosclerotic extent was associated with incremental risk of MACEs during a follow-up of about 3 years. Efforts should be made to determine risk stratification for the management of DM patients with nonobstructive CAD.

## Figures and Tables

**Table 1:** Baseline characteristics.

Characteristic	Value (*N* = 813)
Age (years)	58.9 ± 9.9
Male	391 (48.1%)
Body mass index (kg/m^2^)	26.2 ± 3.6
Cardiac risk factors	
Hypertension	527 (64.8%)
Hypercholesterolemia	442 (54.4%)
Current smoking	197 (24.2%)
Family history of CAD	192 (23.6%)
CCTA findings	
High-risk plaque	18 (2.2%)
CAD-RADS score	
0	190 (23.4%)
1	121 (14.9%)
2	502 (61.7%)
Segment involvement score	1 (1-1)
Segment stenosis score	1 (1-2)
Leiden risk score	2.8 (1.2-4.6)
Medication	
Antiplatelet	245 (30.1%)
Beta blocker	295 (36.3%)
ACEI/ARB	256 (31.4%)
Statin	245 (30.1%)
Calcium channel blocker	145 (17.8%)
Diabetic treatment	
Diet only	160 (19.7%)
Oral hypoglycemic agent	658 (80.9%)
Insulin	116 (14.3%)

Values are mean ± SD or *n* (%). CAD: coronary artery disease; CCTA: coronary computed tomography angiography; ACEI: angiotensin-converting enzyme inhibitor; ARB: angiotensin receptor blocker; CAD-RADS: Coronary Artery Disease-Reporting and Data System.

**Table 2:** Univariate and multivariate analyses of clinical profile and CCTA findings for major cardiovascular events.

	Univariable HR (95% CI)	*p* value	Leiden × CAD	
			Multivariable HR (95% CI)	*p* value
Age (years)	1.04 (1.01-1.07)	**0.009**	1.03 (1.00-1.07)	**0.027**
Male	0.75 (0.43-1.32)	0.325	0.84 (0.47-1.51)	0.556
BMI (kg/m^2^)	1.03 (0.96-1.11)	0.388		
Cardiac risk factors				
Hypertension	1.23 (0.67-2.25)	0.505		
Hypercholesterolemia	1.42 (0.80-2.54)	0.231		
Current smoker	0.95 (0.50-1.82)	0.876		
Family history of CAD	0.69 (0.33-1.42)	0.310		
CCTA findings				
High-risk plaque	11.66 (5.45-24.95)	**<0.001**	10.94 (5.00-23.92)	**<0.001**
Leiden risk score	1.06 (1.00-1.13)	0.055		
Segment involvement score	1.17 (1.00-1.36)	0.048		
CAD severity (Leiden × CAD)				
Normal	Reference		Reference	
Nonobstructive Leiden < 5	1.86 (0.70-5.00)	0.216	1.56 (0.58-4.22)	0.379
Nonobstructive Leiden ≥ 5	4.06 (1.56-10.56)	**0.004**	2.94 (1.11-7.79)	**0.031**

Data in parentheses are 95% confidence intervals. In this analysis, gender and variables with significant (*p* < 0.05) impact on survival at a univariable level entered the multivariable model. HR: hazard rate; CAD: coronary artery disease; CCTA: coronary computed tomography angiography; BMI: body mass index.

## Data Availability

The datasets used and/or analysed during the current study are available from the corresponding author on reasonable request.
